# Effect of Statins on Endothelial Function in Patients With Acute Coronary Syndrome: A Prospective Study Using Adhesion Molecules and Flow-Mediated Dilatation

**DOI:** 10.14740/jocmr1863w

**Published:** 2014-07-28

**Authors:** Ibrahim Altun, Fahrettin Oz, Selda Can Arkaya, Ilknur Altun, Ahmet Kaya Bilge, Berrin Umman, Umit Mutlu Turkoglu

**Affiliations:** a Department of Cardiology, Istanbul School of Medicine, Istanbul University, Istanbul, Turkey; b Department of Biochemistry, Istanbul School of Medicine, Istanbul University, Istanbul, Turkey; c Department of Radiology, Sisli Etfal Training and Research Hospital, Sisli-Istanbul, Turkey

**Keywords:** Statins, Adhesion molecules, Ultrasonography, Acute coronary syndrome, Flow-mediated dilatation

## Abstract

**Background:**

Accumulating evidence suggests that inflammatory mechanisms play a central role in the development, progression and outcome of atherosclerosis. Recent evidence suggests that statins improve anti-inflammatory, anti-thrombotic and endothelial functions, along with their lipid-decreasing effects. We examined the effect of statins on endothelial function using biochemical markers of endothelial dysfunction and brachial artery flow-mediated dilatation (FMD).

**Methods:**

Thirty male patients presenting with acute coronary syndrome (ACS) and 26 age-matched healthy control subjects aged 40 - 60 years who were not on any medication were enrolled in the study. The patient group was started on atorvastatin (40 mg/day) without consideration of their low-density lipoprotein (LDL)-cholesterol levels. Endothelin, sICAM and E-selectin from stored serum samples were measured using commercially available enzyme-linked immunosorbant assays (ELISAs). Endothelial function was assessed using brachial artery FMD.

**Results:**

Prior to statin treatment, E-selectin, sICAM and endothelin levels, endothelial dysfunction markers, were 99.74 ± 34.67 ng/mL, 568.8 ± 149.0 ng/mL and 0.62 ± 0.33 fmol/mL, respectively in the patient group. E-selectin and sICAM levels were significantly higher in the patients than in the control subjects (P < 0.001); however, endothelin levels were not significantly different between groups. Statin treatment significantly reduced E-selectin and sICAM levels (P < 0.001); however, the decrease in endothelin levels was not statistically significant. %FMD values were significantly increased after statin treatment (P = 0.005), and levels of C-reactive protein (CRP), an inflammation marker, were significantly reduced.

**Conclusion:**

Our results indicate that statins play an important role in treatment endothelial dysfunction by reducing adhesion of inflammatory cells.

## Introduction

Accumulating evidence suggests that inflammatory mechanisms play a central role in the development, progression and outcome of atherosclerosis (AS) [1, 2]. Atherosclerotic plaques contain inflammatory cells recruited in response to endothelial damage caused by various stimuli [3, 4]. Inflammatory cells contribute to the development of acute atherothrombotic events, which may cause acute coronary syndrome (ACS) by producing proteolytic enzymes that reduce the mechanical stability of the plaques and increase the risk of rupture [5, 6]. Moreover, ACS is associated with a widespread vascular inflammatory process not confined to the artery responsible for the acute event [7, 8]. Elevated systemic inflammatory markers that persist after the initial event have been shown to predict the recurrence of coronary instability [9]. These observations suggest that in addition to isolated vulnerable plaques, the endothelium may be inflamed and vulnerable for weeks to months after the initial event [6].

Statins reduce serum lipids by inhibiting 3-hydroxy 3-methyl glutaryl-co enzyme A (HMG-CoA) reductase, the rate-limiting enzyme in cholesterol synthesis. Recently, statins have been reported to improve anti-inflammatory, anti-thrombotic and endothelial functions along with their lipid-decreasing effects [10-13]. Accordingly, statins are used widely to decrease short-term mortality and morbidity and to prevent further vascular adverse events in patients with ACS [14-17].

The present study examined the effect of statins on endothelial function in patients diagnosed with ACS. We evaluated endothelial dysfunction using biochemical markers and brachial artery flow-mediated dilatation (FMD) to assess vascular function and to examine the relationship between the two indicators of endothelial dysfunction.

## Patients and Methods

This was a prospective, open-label study. We enrolled 30 male patients presenting with typical chest pain to the emergency unit of the Istanbul Medical Faculty Cardiology Department in Istanbul. Electrocardiography (ECG) and cardiac enzyme evaluations were used to diagnose ACS. The control group consisted of 26 age-matched healthy subjects who were 40 - 60 years old and not on any medication. Control subjects were outpatients who had been referred to us for chest pain, but were free of ischemic heart disease assessed using myocardial perfusion scintigraphy. Age, sex, body mass index and information on the following coronary artery disease risk factors were recorded: hypertension (self-report, blood pressure > 140/90 mm Hg or use of an antihypertensive drug); diabetes mellitus (self-report, fasting glucose > 126 mg/dL or use of oral hypoglycemic agents or insulin); dyslipidemia (self-report, low-density lipoprotein (LDL) > 130 ng/dL, total cholesterol > 200 ng/dL); and nicotine use (within 1 year). All demographic and clinical data were collected prospectively. Creatine kinase-MB isoenzyme (CK-MB), cardiac troponin I (cTnI) and C-reactive protein (CRP) levels were measured at admission. cTnI levels were assessed at 6, 12 and 24 h after admission. The exclusion criteria were immune suppression, glomerular filtration rate < 60 mL/min according to the Cock-Croft-Gault formula, presence of congestive heart failure or a history of coronary artery disease, severe valvular disease, advanced hepatic disease, systemic inflammatory or autoimmune disease, and active malignancy.

We started the patients on atorvastatin (40 mg/day) without consideration of their LDL-cholesterol levels. Control subjects did not receive statin therapy during study period. According to the American National Cholesterol Education Program Adult Treatment Panel 3 (NCEP ADULT P3), LDL-cholesterol should be below 70 mg/dL. Control blood samples were collected from the patients 3 months after they were discharged and endothelial dysfunction and routine biochemical markers were analyzed. The study was approved by the local ethics committees, and all patients provided informed consent.

### ACS

In accordance with the American Heart Association (AHA) and European Society of Cardiology (ESC) guidelines, our patients were diagnosed with ACS if at least two of the following criteria were met: 1) chest pain characteristic of angina (new-onset angina pectoris (AP), or a recent increase in the severity of AP); 2) characteristic changes in the ECG; or 3) a serial rise in the concentration of cardiac enzymes. Furthermore, angiography documenting recent coronary occlusion was considered an ACS event. An elevated cardiac enzyme level in the absence of compatible clinical history was not diagnosed as ACS.

### Laboratory methods

Blood samples were collected from each participant by venipuncture into tubes and centrifuged for 10 min at 2,500 rpm at 4 °C, and the serum samples were aliquoted. Some of the samples were immediately frozen at -80 °C for the investigation of endothelial dysfunction indicators, and the remaining serum was used for analysis of biochemical indicators.

Endothelin (BioMedica Gruppe, Wien, Austria), sICAM (Diaclone Research, Besancon, France) and E-selectin (Diaclone Research) were measured from the stored serum samples using the commercially available enzyme-linked immunosorbent assay (ELISA).

Serum lipids (total cholesterol, triglycerides, high-density lipoprotein (HDL)-cholesterol and LDL-cholesterol), CK-MB and the biochemical indicators were measured using an automated analyzer (Modular DPP Autoanalyzer, Roche Diagnostics, Mannheim, Germany) with commercial kits. A high-sensitivity assay was used to measure CRP (hs-CRP), and cTnI levels were determined using the Abbott AxSYM system analyzer (Abbott Laboratories, Abbott Park, Il, USA) fluorescence immunoassay.

### FMD evaluation using brachial artery high-resolution ultrasound imaging

FMD was measured using ultrasound unit electronic calipers (VIVID 7, General Electric, Waukesha, WI, USA) and a 10-MHz linear array transducer. Briefly, FMD was assessed by measuring the change in brachial artery diameter after 60 s of reactive hyperemia compared with the baseline measurement after deflation of a cuff that had been placed around the forearm and inflated to 50 mm Hg above systolic blood pressure for 5 min. The response of the vessel diameter to reactive hyperemia was expressed as percentage change relative to the diameter immediately before cuff inflation. FMD was expressed as the percentage change from baseline in the brachial artery internal diameter following reactive hyperemia [18, 19]. The interobserver variability of the FMD measurement was 3.5% in our study.

### Statistical analysis

The Statistical Package for the Social Sciences version 10.0 for Windows (SPSS Inc., Chicago, IL, USA) was used to conduct the statistical tests. Unpaired *t*-tests were used for between-group comparisons, and paired Student’s *t*-tests were used for within-group pre- and post-treatment comparisons. Pearson’s correlation coefficient was used to test the correlation between the vasodilation response and indicators of endothelial dysfunction. The results are expressed as means ± standard deviation, and P values < 0.05 were deemed to indicate statistical significance.

## Results

The ASC group consisted of 30 male patients with a mean age of 52.5 ± 9.16 years (range, 34 - 60 years). Of those, 20% had ST-segment elevation (n = 6), 53% had non-ST segment elevation myocardial infarction (n = 16) and 27% had unstable angina pectoris (n = 8). Twenty-one of the ACS patients (70%) had multi-vessel lesions and nine (30%) had a single-vessel lesion. The control group was comprised of 26 healthy adult males with an average age of 49.69 ± 6.23 years (range, 40 - 60 years). The characteristics and routine biochemical parameters according to group, and the maximum cTnI values of the patients at the time of admission, are shown in Table 1.

**Table 1:** Characteristics and Biochemical Markers of Endothelial Dysfunction According to Group (Mean ± SD)

	Control (n = 26)	Patient (n = 30)
Age (years)	49.69 ± 6.23	52.5 ± 9.16
Weight (kg)	74.69 ± 6.10	78.47 ± 10.92
Height (cm)	170.4 ± 4.89	171.7 ± 5.3
BMI (kg/m^2^)	25.6 ± 1.47	26.57 ± 3.13
Systolic BP (mm Hg)	118.3 ± 13.8*	132.3 ± 21.2
Diastolic BP (mm Hg)	81.2 ± 4.1	88.3 ± 13.1
Smoking (%)	86	77
DM (%)	0*	36
HT (%)	0*	42
HL (%)	0*	28
Glucose (mg/dL)	88.19 ± 9.45	93.07 ± 7.78
BUN (mg/dL)	13.9 ± 2.80	15.83 ± 3.46
Creatinine (mg/dL)	0.77 ± 0.12	0.98 ± 0.14
ALT (U/L)	20.77 ± 5.64	31.07 ± 8.56
GGT (U/L)	22.39 ± 11.92	28.50 ± 6.96
Total protein (mg/dL)	7.46 ± 0.45	7.45 ± 0.54
Albumin (mg/dL)	4.58 ± 0.23	3.95 ± 0.51
CK-MB (U/L)	0 - 25*	57.77 ± 18.62
cTnI (ng/mL, baseline)	0 - 0.4*	6.44 ± 2.24
cTnI (ng/mL, highest value)	N/A	33.92 ± 13.98

*P < 0.001, patient vs. control group. BMI: body mass index; BP: blood pressure; DM: diabetes mellitus; HT: hypertension; HL: hyperlipidemia; BUN: blood urea nitrogen; ALT: alanine aminotransferase; GGT: gamma glutamyl transpeptidase; CK-MB: creatine kinase-MB isoenzyme; cTnI: cardiac troponin I.

Pre- and post-treatment lipid values of the patients are shown in Table 2. The administration of atorvastatin significantly reduced total cholesterol (by 13.4%) and LDL-cholesterol (by 19.8%; P < 0.001 for both). Triglyceride levels were reduced by 21.9%; however, this change was not statistically significant (P = 0.074). No significant difference was found between the pre- and post-treatment very-low-density lipoprotein (VLDL)-cholesterol or HDL-cholesterol levels following the administration of atorvastatin (Table 2).

**Table 2:** Pre- and Post-Treatment Lipid Values According to Group (Mean ± SD)

	Control (n = 26)	Patient before treatment (n = 30)	Patient 3 months after treatment (n = 30)	Control 3 months after follow-up (n = 26)
T-cholesterol (mg/dL)	188.0 ± 29.23	194.3 ± 42.48	168.3 ± 25.26*	182 ± 31.3
LDL-cholesterol (mg/dL)	119.1 ± 23.31	123.1 ± 28.94	98.70 ± 16.14*	122 ± 29.2
VLDL-cholesterol (mg/dL)	28.15 ± 13.41	35.67 ± 21.62	31.87 ± 10.56	27.1 ± 16.4
HDL-cholesterol (mg/dL)	40.69 ± 9.25	36.33 ± 8.30	37.70 ± 7.40	41.2 ± 8.5
Triglyceride (mg/dL)	140.8 ± 66.98	173.4 ± 94.27	147.4 ± 59.00**	145 ± 56.8

*P < 0.001, **P < 0.05 pre- vs. post-treatment. LDL: low-density lipoprotein; HDL: high-density lipoprotein; VLDL: very-low-density lipoprotein; T: total.

E-selectin, sICAM and endothelin levels, endothelial dysfunction markers, were 99.74 ± 34.67 ng/mL, 568.8 ± 149.0 ng/mL and 0.62 ± 0.33 fmol/mL, respectively, in the patient group prior to statin treatment. Baseline E-selectin and sICAM levels were significantly higher in patients than in the control group (P < 0.001); however, we found no between-group difference in endothelin levels. Although E-selectin and sICAM levels were significantly reduced after the statin treatment (P < 0.001), the subsequent decrease in endothelin levels was not statistically significant (Table 3). Baseline levels of hs-CRP, an inflammatory marker, were significantly different between the patient and the control groups (P < 0.001). Statin treatment significantly reduced hs-CRP levels from 21.30 ± 39.10 mg/dL to 3.01 ± 0.83 mg/dL (P < 0.05; Table 3).

**Table 3:** Biochemical Marker Levels in the Control Group and in Patients Before and After Statin Treatment (Mean ± SD)

	Control (n = 26)	Patient before treatment (n = 30)	Patient 3 months after treatment (n = 30)	Control 3 months after follow-up
E-selectin (ng/mL)	78.66 ± 24.74	99.74 ± 34.67^a^	89.15 ± 30.50*	81.3 ± 26.3
sICAM-1 (ng/mL)	387.5 ± 113.5	568.8 ± 149.0^a^	490.2 ± 133.0*	422 ± 123.2^b^
Endothelin (fmol/mL)	0.69 ± 0.33	0.62 ± 0.33	0.55 ± 0.20	0.62 ± 0.21
hs-CRP (mg/L)	1.07 ± 0.79	21.30 ± 39.10^a^	3.01 ± 0.83**	2.2 ± 0.81^b^

E-selectin: endothelial selectin; sICAM-1: soluble intercellular adhesion molecule; hs-CRP: high-sensitivity C-reactive protein. ^a^P < 0.001 patient vs. control group at baseline. ^b^P < 0.05 patient vs. control group at the 3-month follow-up. *P < 0.001, **P < 0.05 pre- vs. post-treatment in the patient group.

Brachial artery diameter was measured in the patient and control groups at baseline and after stimulation using the following equation:

%FMD = (Peak hyperemic diameter - baseline diameter/Baseline diameter) × 100

Basal %FMD was not significantly different between the patient and control groups; however, %FMD values were significantly increased in patients with ACS compared with the controls at the 3-month follow up (P < 0.05; Table 4).

**Table 4:** Brachial Artery Diameter Before (Basal) and After Stimulation and %FMD in the Control Group and in the Patients Before and After Statin Treatment (Mean ± SD)

	Control (n = 26)	Patient before treatment (n = 30)	Patient after treatment (n = 30)	Control 3 month follow-up
Basal brachial artery diameter (mm)	3.86 ± 0.43	3.67 ± 0.60	3.87 ± 0.71	3.9 ± 0.92
Artery diameter after stimulation (mm)	4.06 ± 0.44	382 ± 0.59	4.15 ± 0.70*	4.4 ± 0.75
FMD (%)	5.33 ± 5.57	4.86 ± 4.76	7.60 ± 6.23**	6.9 ± 5.6

*P < 0.01, **P < 0.05, patient group pre- vs. post-stimulation. FMD: flow-mediated dilatation.

E-selectin and endothelin levels and %FMD were not significantly different between groups at 3 months after the onset of treatment. However, sICAM-1 and hs-CRP were significantly lower in the control than in the ACS group (Table 3).

**Figure 1:**
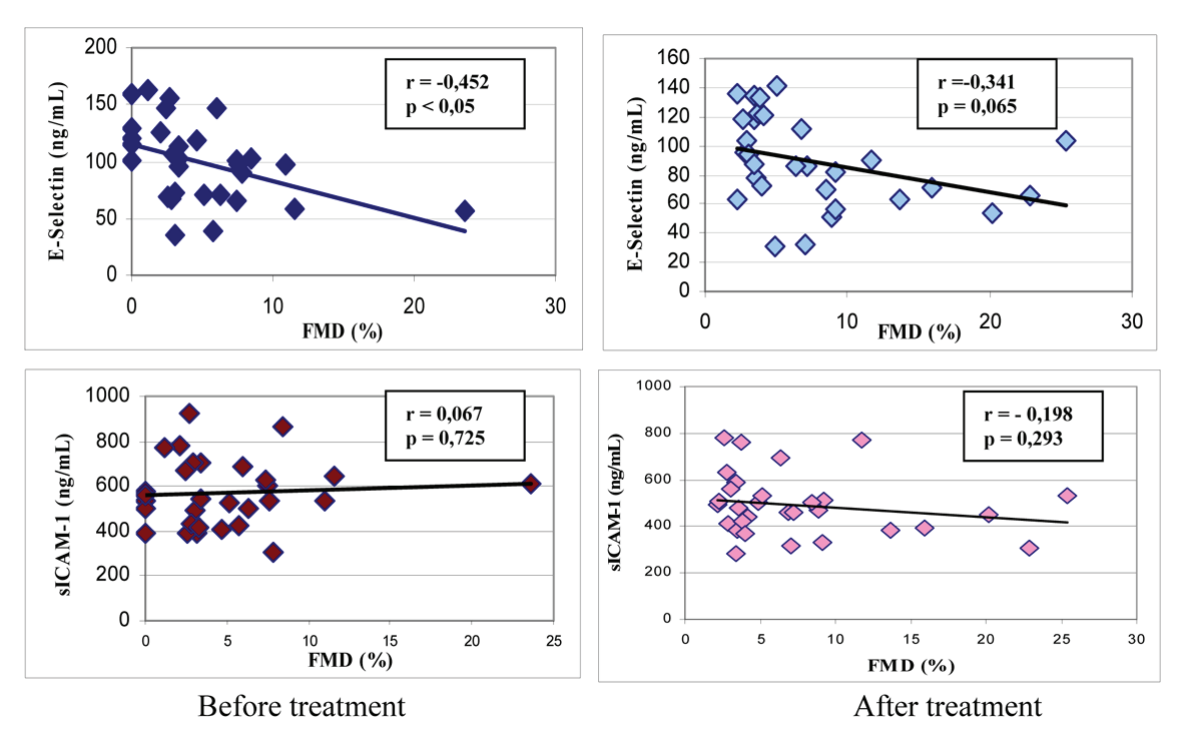
Correlations between %FMD and E-selectin and sICAM-1 levels in patients before and after treatment (mean ± SD).

We found a significant negative correlation between FMD and baseline E-selectin levels in the patients; however, the correlations between FMD and the other parameters were not significant (Fig. 1). We found a significant positive correlation between E-selectin and sICAM, total cholesterol, and LDL-cholesterol levels (Table 5).

**Table 5:** Correlation Between E-Selectin and FMD and Biochemical Markers of Endothelial Dysfunction Before and After Statin Treatment in the Patients (Mean ± SD)

E-selectin	Before treatment		After treatment	
	Correlation factor (r)	P value	Correlation factor (r)	P value
%FMD change	-0.452	0.012*	-0.341	0.065
sICAM-1	0.434	0.016*	0.278	0.142
Endothelin	0.132	0.488	0.297	0.111
hs-CRP	0.078	0.682	0.099	0.601
Total-cholesterol	0.367	0.046*	0.347	0.060
LDL-cholesterol	0.385	0.036*	0.429	0.018*
Triglyceride	0.058	0.763	-0.101	0.596

*P < 0.05. sICAM-1: soluble intracellular adhesion molecule-1; hs-CRP: high-sensitivity C-reactive protein; LDL: low-density lipoprotein; FMD: flow-mediated dilatation.

## Discussion

We found that atorvastatin therapy improved endothelial function in patients with ACS as assessed by FMD and adhesion molecule levels. Our results showed that the baseline plasma levels of the adhesion molecules were significantly higher and %FMD was significantly lower in patients with ACS compared with the control group. However, treatment with atorvastatin for 3 months significantly increased %FMD and significantly reduced plasma E-selectin, sICAM and hs-CRP levels in the patients. Moreover, we found that atorvastatin (40 mg/day) significantly reduced total (13.4%) and LDL-cholesterol levels (19.8%) early in the course of the therapy. However, statin treatment did not significantly change triglycerides, VLDL- or HDL-cholesterol levels.

Statins reduce cholesterol synthesis through inhibition of the rate-limiting enzyme, HMG-CoA reductase, and are prescribed widely for their serum-lipid-lowering effect. Prospective clinical trials have demonstrated that statins reduce LDL-cholesterol and decrease morbidity and mortality rates, coronary artery disease, in particular [20-22]. However, the beneficial effect of statins occurs early in the course of therapy. Recent experimental evidence has shown that statins have anti-inflammatory, anti-thrombogenic and endothelium-dysfunction-correcting effects in addition to their lipid-lowering effects [11-14]. Leukocytes and platelet interactions with endothelial cells play a central role in the development of the inflammation that causes vascular disease. Accumulating evidence suggests that statins decrease the number of inflammatory cells in atherosclerotic lesions and reduce adhesion molecules [22-25]. Moreover, fluvastatin has been shown to inhibit the expression of adhesion molecules in a human monocyte cell line. Statins protect the ischemic myocardium in normocholesterolemic and diabetic animals by attenuating P-selectin expression and leukocyte adhesion. However, the relationship between AS and adhesion molecules in humans is controversial [26, 27]. Two previous studies found that statin pretreatment decreased the expression of adhesion molecules induced by tumor necrosis factor alpha (TNFα), interleukin 1 (IL 1), or lipopolysaccharide (LPS) [26, 27]. In contrast to previous reports of the beneficial effects of statins on adhesion molecules, Schmidt et al [25] found that statins increased ICAM-1, vascular cell adhesion molecule 1 (VCAM-1) and TNF? expression by human vascular endothelial cells. Rauch et al [26] found no effect of statins on fibrinogen, sL-selectin, sP-selectin and sICAM-1 levels in patients with hypercholesterolemia after more than 3 months of treatment. In view of these conflicting results, we investigated the effect of atorvastatin on adhesion molecule expression in patients with ACS to clarify the effect of statins on adhesion molecule expression.

We found that statin treatment for 3 months reduced sE-selectin and sICAM-1 levels in patients with ACS. Furthermore, we found a significant decrease in CRP levels, an inflammation marker. The brachial artery ultrasound scan revealed that the vasodilatory response improved with statin treatment. Examination of the association between plasma sE-selectin and sICAM-1 levels and the vasodilator response revealed a negative correlation between sE-selectin and %FMD, but no significant relationship between sICAM-1 and %FMD. sE-selectin is found primarily in the endothelium, whereas sICAM-1 is located in multiple cell types including leukocytes, fibroblasts and the intimal smooth muscle cells found in atherosclerotic lesions [28]. Thus, a negative correlation between endothelial sE-selectin and the vasodilator response seems reasonable. However, it is possible that we failed to find evidence supporting sICAM-1as a plasma marker of endothelial dysfunction because our sample size was small, our follow-up period was relatively short and we used a low dose of atorvastatin. Moreover, we measured the plasma adhesion molecules as soluble antigens and the origin of the soluble forms is not clear. Increased production of soluble forms could be attributed to increased transcription or enhanced proteolytic cleavage from the cell surface. Due to changes in the endothelium and the E-selectin and ICAM-1 expression in the cells connected to the membrane surface in the bloodstream and the effect of statins on these proteins on the cell surface is limited.

We found that atorvastatin therapy improved endothelium-dependent vasodilatation measured by %FMD. Our results are consistent with a previous report that pravastatin treatment for 6 months improved endothelium-dependent coronary vasodilatation in patients with hypercholesterolemia [29]. A previous study in patients with AS found that treatment with lovastatin for 6 months significantly improved endothelium-mediated responses in the coronary artery [30]. A number of mechanisms have been proposed to mediate this effect. We found that statins play a central role in reducing circulating levels of the adhesion molecules ICAM-1 and E-selectin. Our findings suggest that statins reduce platelet and leukocyte adhesion and improve endothelial function. However, a study in patients with coronary artery disease and mildly elevated cholesterol levels found no difference between placebo and simvastatin in endothelium-dependent vasodilatation following treatment for 6 months [31]. The discrepancy between our results and those of the previous study may be explained by the fact that their baseline levels of total and LDL-cholesterol were lower than ours, the baseline AS was less severe and baseline endothelial dysfunction was relatively mild compared with that in studies showing improved endothelial function.

The present study had several limitations including a short follow-up period, a relatively small sample size, and the open label, non-randomized design. Moreover, our patients were allowed to use cardioprotective drugs such as angiotensin converting enzyme inhibitors, angiotensin receptor blockers, acetyl salicylic acid and beta-blockers; this may have had additional positive effects on endothelial function.

### Conclusion

Our findings support the pleiotropic effects of the lipid-lowering statin, atorvastatin. Our results suggest that statins play an important role in the treatment of endothelial dysfunction and in decreasing the adhesion of inflammatory cells to the endothelium in patients with ACS.
